# Differential impact of glycemic control and comorbid conditions on the neurophysiology underlying task switching in older adults with type 2 diabetes

**DOI:** 10.18632/aging.204129

**Published:** 2022-06-17

**Authors:** Christine M. Embury, Grace H. Lord, Andjela T. Drincic, Cyrus V. Desouza, Tony W. Wilson

**Affiliations:** 1 Institute for Human Neuroscience, Boys Town National Research Hospital, Boys Town, NE 68010, USA; 2 Department of Psychology, University of Nebraska Omaha, Omaha, NE 68182, USA; 3 Department of Internal Medicine, Division of Diabetes, Endocrinology and Metabolism, University of Nebraska Medical Center, Omaha, NE 68198, USA

**Keywords:** magnetoencephalography, cognitive control, executive functioning, glycemic control, comorbidities

## Abstract

Type 2 diabetes is known to negatively affect higher order cognition and the brain, but the underlying mechanisms are not fully understood. In particular, glycemic control and common comorbidities are both thought to contribute to alterations in cortical neurophysiology in type 2 diabetes, but their specific impact remains unknown. The current study probed the dynamics underlying cognitive control in older participants with type 2 diabetes, with and without additional comorbid conditions (i.e., cardiovascular disease, nephropathy, peripheral neuropathy, retinopathy), using a task switching paradigm and a dynamic functional brain mapping method based on magnetoencephalography (MEG). We hypothesized that neural dynamics would be differentially impacted by the level of glycemic control (i.e., diabetes itself) and the burden of additional comorbid conditions. Supporting this hypothesis, our findings indicated separable, but widespread alterations across frontal, parietal, temporal and cerebellum regions in neural task-switch costs in type 2 diabetes that were differentially attributable to glycemic control and the presence of comorbid conditions. These effects were spatially non-overlapping and the effects were not statistically related to one another. Further, several of the effects that were related to the presence of comorbidities were associated with behavioral performance, indicating progressive deficits in brain function with extended disease. These findings provide insight on the underlying neuropathology and may inform future treatment plans to curtail the neural impact of type 2 diabetes.

## INTRODUCTION

Type 2 diabetes is a chronic metabolic disorder characterized by progressive insulin resistance that is often accompanied by a collection of comorbid conditions, many of which are also progressive. Common comorbid conditions include retinopathy, micro- and macro-vascular disease, peripheral neuropathy, and nephropathy. Previous studies have also found significant cognitive and neural deficits in people with type 2 diabetes. Specific cognitive domains affected include memory, attention, and executive functioning, with effect sizes ranging from 0.25–0.5 [1, 2], although moderate to large effect sizes have been found in people over 65 years-old [3], suggesting these decrements increase with aging. Studies focusing on the neurological impact of type 2 diabetes have shown widespread impact, including deficits in grey matter volume and white matter integrity [1, 4–7], with some studies linking these declines to cognitive outcomes and an increased risk for dementia [6, 8]. Likewise, functional imaging studies have shown alterations in resting state functional connectivity and task-based neural responses across widespread networks, including the default mode and frontal-parietal networks [9–12]. Increased numbers of white matter hyperintensities and greater cerebrovascular damage have also been noted in people with type 2 diabetes [1, 10, 13].

Executive functioning is one cognitive domain particularly affected in type 2 diabetes [1, 2]. One task known to tap executive function is task switching, which requires participants to flexibly adjust goal sets and behavior to match contextual cues [14]. Behaviorally, switching rule sets requires longer reaction times relative to stay or no switch trial types, revealing a consistent behavioral switch cost [15]. These paradigms have generally been found to elicit activity across the frontal-parietal and cingulo-opercular networks [16]. Previous EEG studies have also shown greater alpha, beta and theta recruitment in switch, relative to same set trials, reflecting neural switch costs [17, 18]. An MEG study has also found a role for gamma in switch tasks, where neural switch costs were greater across frontal-parietal and cingulate regions [19]. However, such neural dynamics have not yet been probed in the context of type 2 diabetes.

While studies have shown that type 2 diabetes affects executive function, whether the driving force behind these deficits is primarily related to dysglycemia or the increased number of comorbid conditions in those with type 2 diabetes remains to be elucidated. Studies have found mediating effects of high body mass index, age, microvascular damage, and hypertension, as well as glycated hemoglobin (HbA_1c_) and other metrics of glycemic control on the cognitive decrements associated with type 2 diabetes [1, 12, 20]. Insulin resistance, a hallmark of the disease, has a direct impact on the brain. Insulin receptors are found across the cortex and subcortical structures including the hippocampus, hypothalamus, and amygdala [21, 22], and insulin signaling has been implicated in several cognitive processes, most notably episodic memory performance [22]. Both glycemic clamp and insulin administration studies show immediate improvement in cognition with normalized glucose levels, especially within the memory domain [23–25].

In the current study, we used a task switching paradigm [19] and MEG to probe the neural dynamics and circuitry serving this essential component of executive function in a cohort of older adults with type 2 diabetes, about half of whom had common comorbidities. We hypothesized that both glycemic control and comorbid conditions would alter oscillatory activity across relevant networks, including occipital, parietal, and prefrontal regions, suggesting an accelerated aging phenotype with worse glycemic control and the presence of comorbidities. We further posited that the effects of glycemic dysregulation and comorbidities would be separable and that some effects would relate to behavioral switch costs.

## RESULTS

### Demographic, behavioral and disease status results

**Figure 1:**
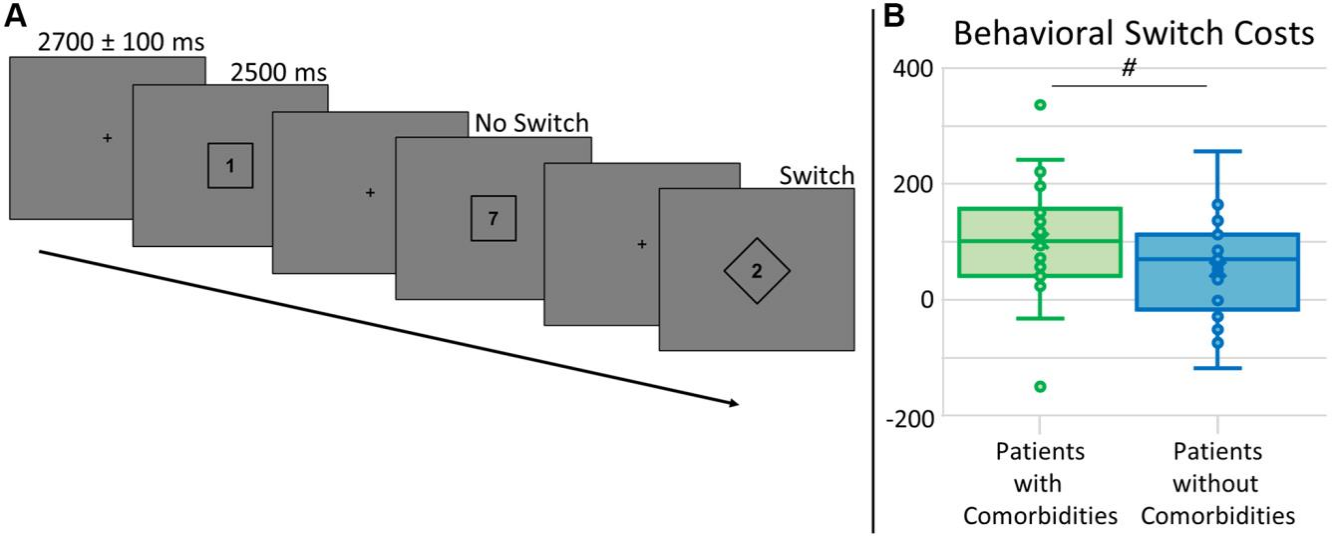
**Task switch paradigm and behavioral switch costs.** (**A**) A fixation cross was presented for 2700 ± 100 ms, followed by a number (possible values: 1–4, 6–9) surrounded by a square or diamond, indicating the rule set for that trial, presented for 2500 ms. For trials with a number within a square, participants had to respond by button press as to whether the number was below (index finger) or above (middle) 5. For trials with a number within a diamond, participants had to answer whether the number was odd (index finger) or even (middle) by button press. Trials were pseudorandomized such that 50% of trials were the same rule set as the previous trial (No Switch) and 50% were a different rule set from the previous trial (Switch). All analyses were relative to the difference between these conditions, or switch costs. (**B**) Accuracy did not differ by group (not shown), but there was a trend for group differences in behavioral switch costs (*t*_46_ = 1.79, *p* = 0.080), where those with type 2 diabetes and additional comorbidities (shown in green) had longer reaction times relative to those without comorbidities (shown in blue). ^#^ denotes 0.050 < *p* < 0.100.

Four participants had to be excluded due to MEG technical issues or artifactual MEG data. Two additional participants were excluded due to behavioral performance at or near chance levels on the task switching paradigm (see Figure 1A). This resulted in a sample of 48 participants (25 with significant comorbidities, 23 without). See demographics and blood panel values in Table 1. Groups did not differ by age (*p* = 0.497), sex (*p* = 0.951), race (*p* = 0.845), education (*p* = 0.132), handedness (*p* = 0.502), BMI (*p* = 0.180), and HbA_1c_ (*p* = 0.205). Of those with comorbidities, 11 had nephropathy (GFR 45–60, ACR 41.45–375), five had retinopathy (mild), 14 had peripheral neuropathy (generally mild, limited to specific extremities), and six had cardiovascular disease (three stents, three bypasses), with nine (36%) of these participants having two or more of these conditions. Participants performed well, with an average accuracy of 95.5 ± 3.6%, average reaction time of 1527.67 ± 216.02 ms, and an average behavioral switch cost, or the difference in reaction times between conditions, of 79.27 ± 96.37 ms. Accuracy did not differ between groups (*t*_46_ = −0.88, *p* = 0.381), but there was a trend for behavioral switch costs differences by group (*t*_46_ = 1.79, *p* = 0.080, see Figure 1B), as those without comorbidities were less affected by the switch.

**Table 1:** Task switch: demographics and laboratory tests.

	**Type 2 diabetes with comorbidities**	**Type 2 diabetes without comorbidities**
***N***	25	23
**Age (years)**	64.0 ± 4.8	62.7 ± 5.1
**Sex**	10 Males; 15 Females	8 Males; 15 Females
**Handedness**	24 Right; 1 Left	21 Right; 2 Left
**Disease duration (years)**	14.1 ± 2.0	10.0 ± 7.5
**HbA_1c_ (mmol/mol)**	59 ± 11.7	55 ± 9.2
**HbA_1c_ (%)**	7.52 ± 1.07	7.16 ± 0.84
**Creatinine (mg/dL)**	0.84 ± 0.22	0.83 ± 0.23
**Glucose (mg/dL)**	119.52 ± 22.75	121.91 ± 41.01
**Albumin/Creatinine (ugAL/mgCR)**	45.08 ± 81.41	17.17 ± 13.40
**Thyroid-stimulating hormone (mcIU/mL)**	2.72 ± 1.72	1.77 ± 0.86
**B12 (pg/mL)**	451.45 ± 361.81	343.08 ± 222.00

Values depict means ± SD.

### Sensor level results

**Figure 2:**
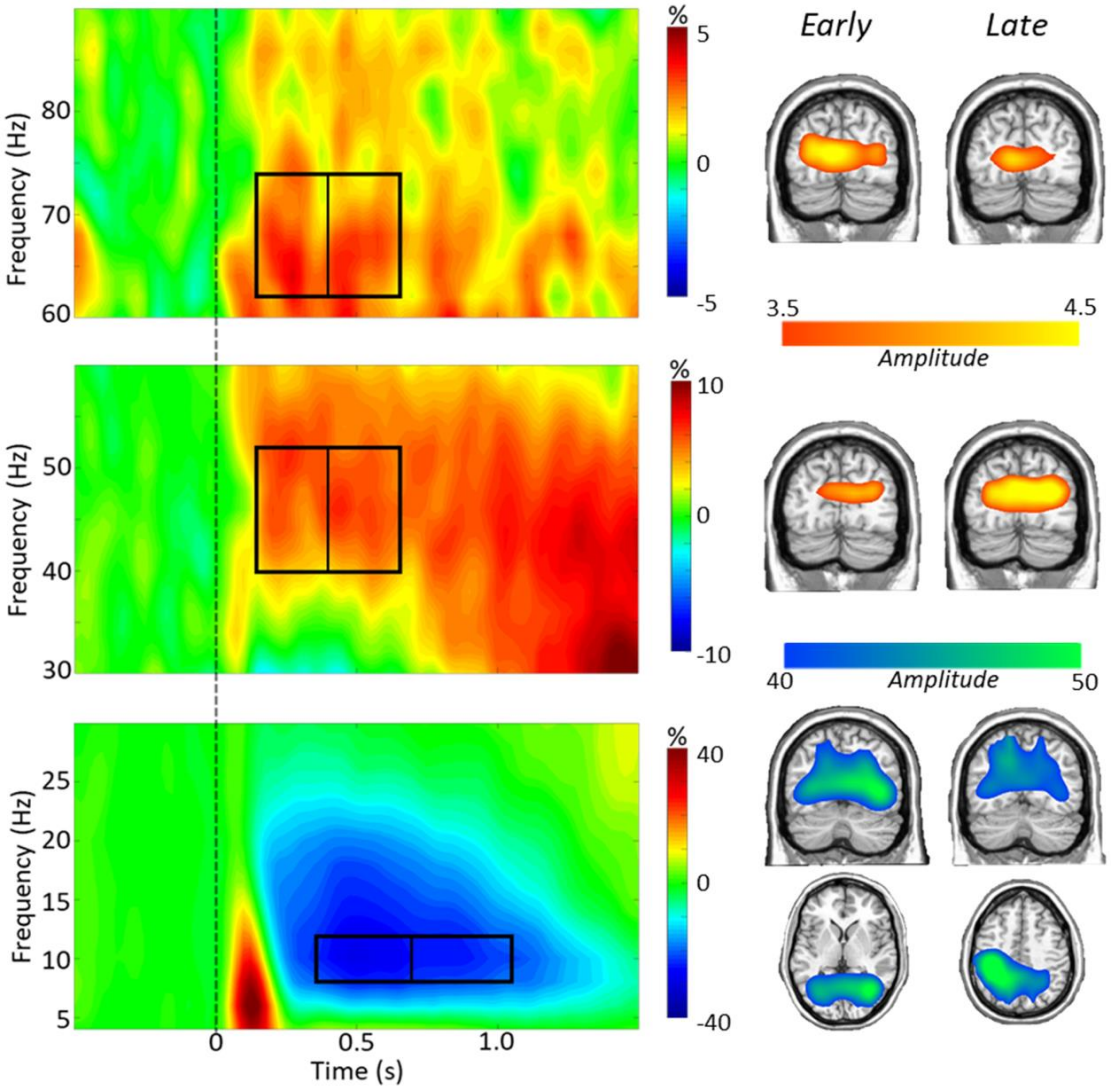
**Task switch spectrograms.** Left: Grand-averaged time-frequency spectrograms are shown, derived from representative parieto-occipital MEG sensors. Time is shown on the x-axis in seconds, frequency is shown on the y-axis in Hz. Spectrograms are shown from 4–30 Hz (bottom), 30–60 Hz (middle), and 60–90 Hz (top). The colors reflect power increases (red) and decreases (blue) relative to the baseline, with the scale bar shown to the right of each time-frequency plot. Time-frequency windows for source imaging (beamforming) were derived from statistical analysis of the sensor-level data across all participants (*ps* < 0.001). A clear alpha (8–12 Hz) decrease can be seen throughout the task period, as well as a lower gamma (40–52 Hz) and a higher gamma (62–74 Hz) synchronization during the task period. Right: Grand average images from each time-frequency bin show the alpha dynamics in parietal and occipital regions, and gamma dynamics largely restricted to bilateral occipital cortices.

To identify significant oscillatory responses for dynamic imaging, *t*-tests between the active and baseline periods per time-frequency bin were computed across all participants and both conditions, which were then corrected for multiple comparisons using nonparametric permutation testing. Significant time-frequency windows were found in alpha (8–12 Hz) from 350–1050 ms and in gamma (40–52 Hz, 62–74 Hz) from 150–650 ms (*p* < 0.001; Figure 2). To study early and late dynamics of task switch processing, alpha was imaged from 350–700 and 700–1050, while both gamma frequency bins were imaged from 150–400 and 400–650 ms. To identify the anatomical origins of these oscillatory responses, images per response window were grand averaged across conditions and participants and this revealed that alpha responses originated in parieto-occipital regions throughout the task period, while both low (40–52 Hz) and high (62–74 Hz) gamma were strongest in bilateral occipital cortices (Figure 2).

### Source level results

#### Neural switch cost differences by patient groups

**Figure 3:**
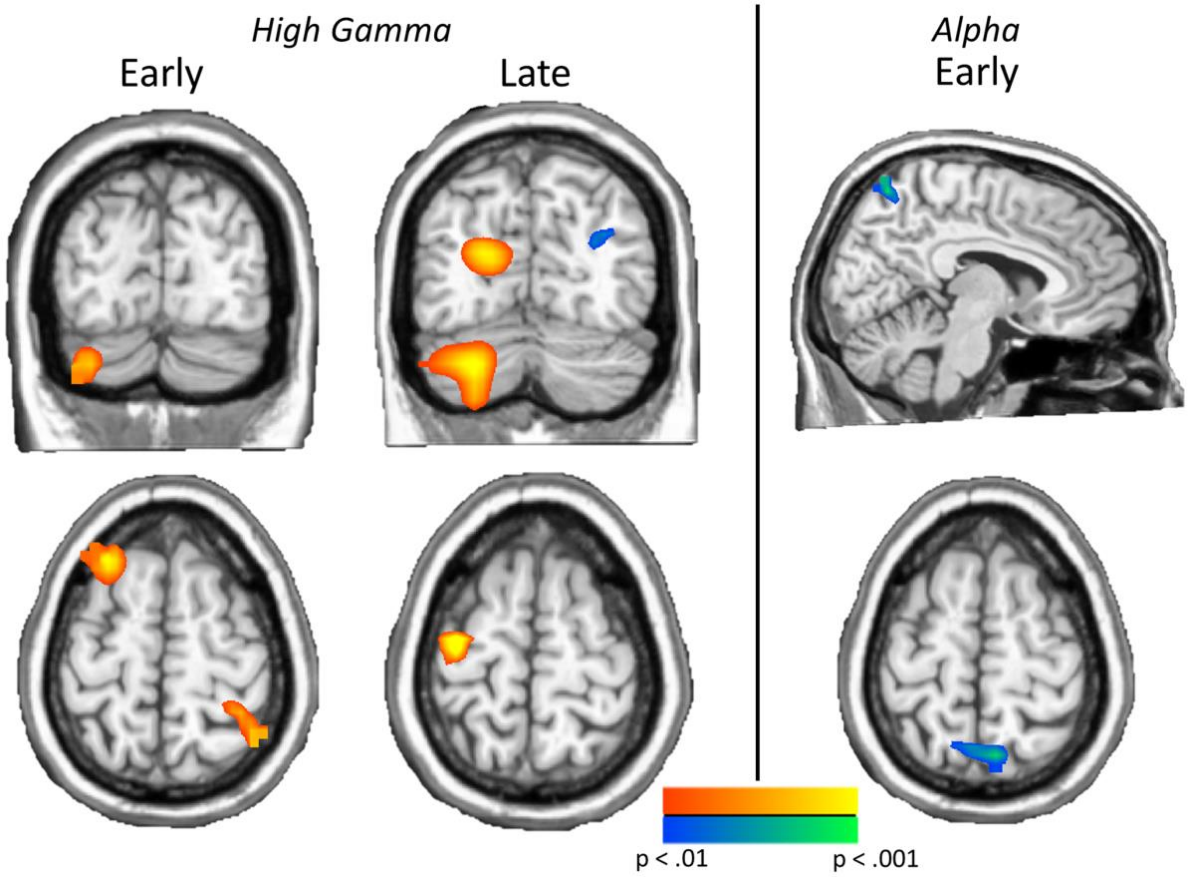
**Neural switch costs differ by comorbidity status.** Left: Significant group effects on neural switch costs were found in both time bins of high gamma, including the left cerebellum, left inferior (not shown) and superior frontal regions and the right parietal cortex in the early high gamma window, and left precentral, left occipital, left cerebellum, and right parieto-occipital regions in late high gamma activity. Right: Greater neural switch costs in early bin parietal alpha activity were found in type 2 diabetes patients with additional comorbidities, relative to those without comorbidities. These effects show the impact of comorbidities on neural switch costs across the spectrum. Images are thresholded from *p* < 0.01 to *p* < 0.001, with a minimum cluster size of 20 4 mm^3^ voxels.

In order to examine neural switch cost effects, whole brain subtraction maps were computed (switch – no-switch) and these maps were compared using independent-sample *t*-tests to identify group effects (patients with comorbidities vs. patients without comorbidities). As shown in Figure 3, our results revealed high gamma (62–74 Hz) differences from 150–400 ms in the left cerebellum (*t*_45_ = 3.32, *p* = 0.002), left inferior frontal (*t*_45_ = 3.78, *p* < 0.001), left superior frontal (*t*_45_ = 3.70, *p* < 0.001), and right inferior parietal (*t*_45_ = 3.76, *p* < 0.001) cortices. In the later 400–650 ms window, high gamma group differences were found in the left precentral (*t*_45_ = 4.55, *p* < 0.001), left occipital (*t*_45_ = 3.53, *p* < 0.001), left cerebellum (*t*_45_ = 3.68, *p* < 0.001), and right parieto-occipital (*t*_45_ = −3.03, *p* = 0.004) regions. In all cases except the right parieto-occipital, the group with comorbidities exhibited stronger high gamma oscillations during task performance (Figure 3). No group differences were detected in low gamma. As per alpha, a significant group difference in neural switch cost was found in the right parietal lobe during the 350–700 ms time window (*t*_44_ = −3.42, *p* = 0.001) such that those with comorbidities had greater switch costs in this region, while no group differences were found in the later alpha time window. Notably, none of these effects were associated with HbA_1c_ level (*p*s from 0.436 to 0.797).

### Neuro-behavioral switch cost associations

**Figure 4:**
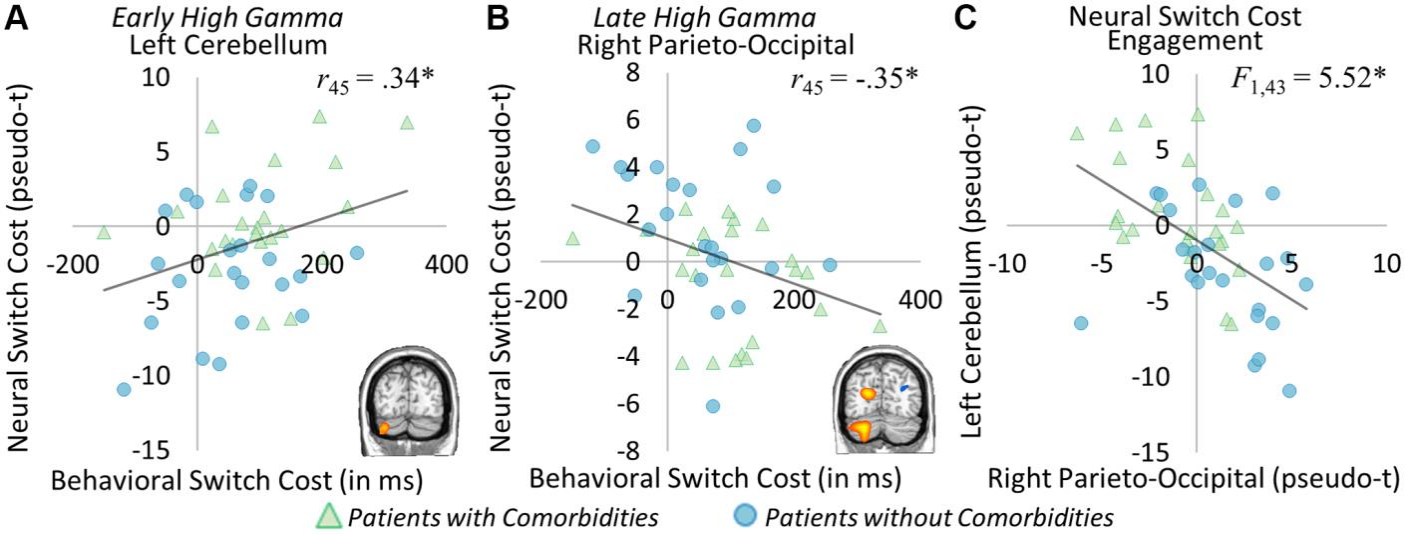
**Neural and behavioral switch cost correlations.** Significant correlations between neural and behavioral switch costs were apparent in the early high gamma left cerebellar peak ((**A**) *r*_45_ = 0.34, *p* = 0.020) and the late high gamma right parieto-occipital peak ((**B**) *r*_45_ = −0.35, *p* = 0.016). In the left cerebellum, greater neural switch costs were associated with greater behavioral switch costs, while in the right parieto-occipital lower neural switch costs were related to greater behavioral switch costs. This distinct pattern of neural responses leading to differential behavioral outcomes was further investigated, and this analysis showed group-specific recruitment of each region ((**C**) *F*_1,43_ = 5.52, *p* = 0.024), whereby those with comorbid conditions (shown in green) had greater switch costs in left cerebellum, while those without comorbidities (shown in blue) had greater switch costs in the right parieto-occipital region. ^*^p < 0.05, ^**^p < 0.01, ^***^p < 0.001.

Peak values were extracted from each of the significant group difference clusters and correlations with behavior were computed. Significant correlations across both groups were found between neural and behavioral switch costs. Specifically, in the early high gamma window, a significant relationship between the left cerebellar peak and behavioral switch cost was apparent (*r*_45_ = 0.34, *p* = 0.020; Figure 4A), such that as the behavioral switch cost became larger, so too did the neural switch costs in this region. In the later high gamma window, a significant inverse relationship between the right parieto-occipital peak and behavioral switch cost emerged (*r*_45_ = −0.35, *p* = 0.016), as increased behavioral switch cost was associated with reduced neural switch costs (Figure 4B). Because of the differing trajectories of these responses across time, we probed the relationship between neural switch costs in these two regions and found a significant reciprocal effect, whereby increased switch costs in one region tracked with decreased switch costs in the other. To determine whether this reciprocal pattern was related to group, a repeated-measures ANCOVA was computed to determine the relationship between neural and behavioral switch costs and group identity. In this model, a significant interaction between neural switch costs at each peak and behavioral switch cost was again apparent (*F*_1,43_ = 4.31, *p* = 0.044). Further, controlling for the effect of behavior, there was a significant interaction of peak by group, such that each group tended to have greater neural switch costs in one peak over the other to complete the task (*F*_1,43_ = 5.52, *p* = 0.024, see Figure 4C). In this case, participants with type 2 diabetes and comorbidities tended to have higher switch costs in the early bin left cerebellum peak and lower switch costs in the late bin right parieto-occipital peak, whereas participants with type 2 diabetes and no comorbidities tended to have higher switch costs in the late bin right parieto-occipital peak and lower switch costs in the early bin left cerebellum peak. No significant interaction of group by behavioral switch costs (*F*_1,43_ = 0.12, *p* = 0.735), nor peak by group by behavioral switch cost (*F*_1,43_ = 0.04, *p* = 0.851) was found. These findings suggest a differential impact of comorbidities on neural oscillatory responses serving higher order cognition in type 2 diabetes, but group membership did not specifically predict behavioral outcomes.

### Neural switch cost correlations with clinical metrics

**Figure 5:**
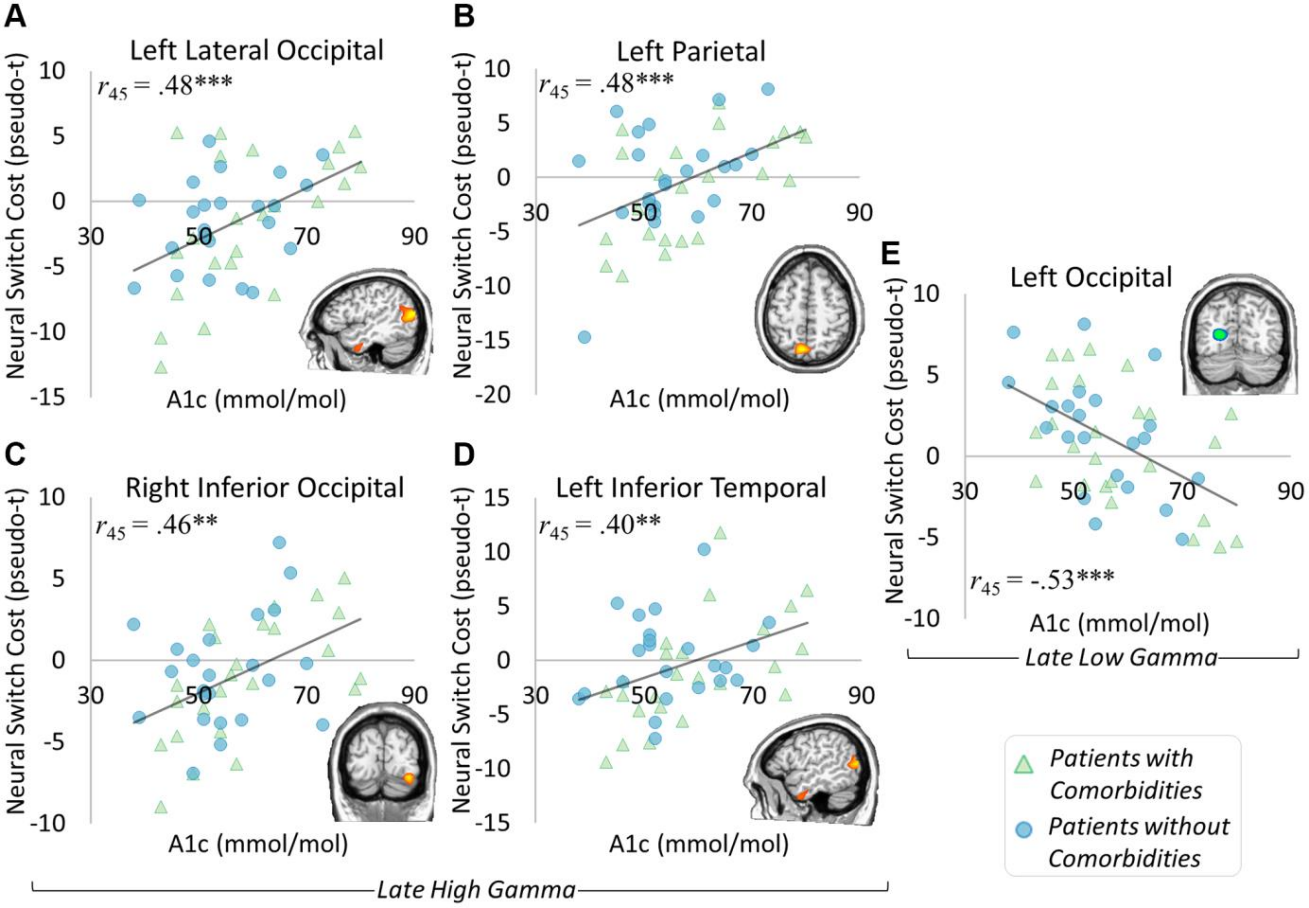
**Glycemic control and neural switch cost associations.** Significant correlations between neural switch cost dynamics and glycemic control level were apparent in the late high gamma and late low gamma windows. Specifically, gamma responses in the left lateral occipital ((**A**) *r*_45_ = 0.48, *p* < 0.001), left parietal ((**B**) *r*_45_ = 0.48, *p* < 0.001), right inferior occipital ((**C**) *r*_45_ = 0.46, *p* = 0.001), and left inferior temporal ((**D**) *r*_45_ = 0.40, *p* = 0.005) regions exhibited greater neural switch costs in the context of higher HbA_1c_ levels. Interestingly, greater neural switch costs were associated with lower HbA_1c_ levels in the left occipital region’s late low gamma response ((**E**) *r*_45_ = −0.53, *p* < 0.001). These results show a differential impact of glycemic control level on neural switch costs, with separable effects from those seen in differences by comorbidity status. ^*^p < 0.05, ^**^p < 0.01, ^***^p < 0.001.

To investigate the relationship between switch costs and glycemic control, we ran whole brain correlations on the neural switch cost maps. Several areas correlated with HbA_1c_ level in the late high gamma window including the left parietal (*r*_45_ = 0.48, *p* < 0.001), left lateral occipital (*r*_45_ = 0.48, *p* < 0.001), right inferior occipital (*r*_45_ = 0.46, *p* = 0.001), and left inferior temporal (*r*_45_ = 0.40, *p* = 0.005) regions. These effects were such that as HbA_1c_ increased, high gamma switch costs also increased in each region (Figure 5A–5D). A significant correlation with HbA_1c_ was also apparent in the early low gamma window in the left occipital cortex (*r*_45_ = −0.52, *p* < 0.001), such that as HbA_1c_ levels increased, low gamma switch costs decreased (Figure 5E). None of these effects differed by comorbidity distinction (*p*s from 0.386 to 0.965). These results suggest largely distinct effects of glycemic dysregulation and the presence of comorbid conditions.

## DISCUSSION

We found that glycemic dysregulation and the presence of comorbid conditions have largely distinct effects on the neurophysiological activity underlying task switching in a sample of older adults with type 2 diabetes. The impact of comorbid conditions was largely an increase in behavioral switch costs, as well as an increase in the strength of oscillations in the high gamma range across multiple brain regions, including the left superior and inferior frontal regions, left precentral gyrus, left cerebellum, and bilateral occipital areas. Comorbidities were also associated with aberrations in right parietal alpha oscillations. Such accentuated neural switch costs across frontal-parietal network regions suggests that comorbidities may affect hub regions involved in cognitive control, as well as cerebellar activity that has been linked to error monitoring and conflict resolution [26, 27], all of which are subprocesses contributing to task switching performance. Similarly, inhibitory control has also been shown to be diminished in type 2 diabetes [28, 29], so it follows that the underlying neural correlates would be impacted. Regarding glycemic control, increased HbA_1c_ levels were associated with larger neural switch costs (i.e., stronger neural oscillations) in the high gamma range within superior parietal, lateral occipital, inferior temporal, and ventral occipital cortices, as well as smaller neural switch costs in medial occipital cortex in the lower gamma range. Thus, the brain regions most strongly affected by glycemic dysregulation were largely independent of those associated with significant comorbidities. Below, we discuss the implications of these findings for understanding the neural aberrations often seen in those with type 2 diabetes, especially regarding how these relate to decrements in task switching.

Some of our key findings were that comorbid conditions were associated with increases in the strength of high gamma oscillations across the left superior and inferior frontal regions, left precentral gyrus, left cerebellum, and bilateral occipital areas, as well as right parietal alpha. The frequency specificity of these findings likely reflects the underlying processes directly affected by comorbid conditions associated with type 2 diabetes. Specifically, frontal and occipital gamma responses have previously been implicated in tasks probing executive functioning and cognitive control [30–32]. These gamma responses have also been implicated in a wide range of functions including attention, working and long-term memory, and feature binding [33, 34]. Parietal alpha has an important role in the dorsal attention network, where it has been linked to the top-down direction of attention toward task goals [35]. While the widespread nature of effects also suggests a more global impact of comorbid conditions in type 2 diabetes, the relatively large number of findings in the gamma band may suggest effects on specific mechanisms underlying inhibitory processes, such as gamma-aminobutyric acid (GABA) signaling. GABA is one of the key receptors known to be downregulated in the insulin resistant brain [36]. Gamma dynamics are thought to regulated by local GABA-ergic inhibitory circuitry [37, 38]. Indeed, in previous studies in people with type 2 diabetes, GABA levels in occipital and prefrontal cortices were shown to be significantly altered, with some effects directly associated with cognitive task performance [39–42]. These alterations in GABA could be a critical mechanism underlying many of our findings in the current study, as many effects in our task switching paradigm were in the gamma range. Future studies should further examine GABA-gamma links in this population using combined MEG and MRI-based GABA spectroscopy.

We also showed significant associations between neural and behavioral switch costs. In this case, greater neural switch costs in the left cerebellum’s early gamma response was associated with greater behavioral switch costs, while smaller neural switch costs in the right parieto-occipital cortex’s late gamma response was associated with greater behavioral switch costs. Further, these relative switch costs were separable by group, reflecting the unique impact of comorbidities in type 2 diabetes. Of note, participants with comorbidities generally exhibited more aberrant activity in the left cerebellum, where greater neural switch costs led to greater behavioral switch costs. On the other hand, participants without comorbid conditions tended toward the more compensatory pattern, where increased right parieto-occipital activity led to less behavioral switch costs. These patterns of findings suggest that the additional comorbidities lead to an inefficient system underlying cognition and behavior. Previous studies have also shown that the presence of comorbidities, particularly those affecting vascular integrity, have strong detrimental effects on the brain and cognition [1, 3, 43, 44], albeit without the frequency specificity of neural effects we were able to discern in the current study.

Importantly, the peak neural differences between those with and without comorbidities did not spatially overlap with those that varied based on level of glycemic control, as measured by HbA_1c_. Further, comorbidity status associated effects did not relate to HbA_1c_ level and glycemic control findings did not differ by comorbidity status. Instead, neural switch costs in several other brain regions were found to significantly correlate with HbA_1c_, including the left occipital cortex in the late low gamma window and left lateral occipital, left parietal, left inferior temporal, and right inferior occipital regions in late high gamma window. Higher HbA_1c_ levels were associated with larger neural switch costs in the high gamma peaks, and weaker neural switch costs in the low gamma range. Higher HbA_1c_ has been previously shown to have worse cognitive outcomes [45, 46], although some studies suggest that the relationship is weak, only accounting for about 10% of the variance in measured decrements [47]. While it is generally true that worse glycemic control generally leads to greater incidence of comorbidities, from our findings, the effects of glycemic control and the presence of comorbid conditions differentially impact the brain and behavior. Further, neural switch cost differences by comorbidity status related more closely to behavior, where in general, greater neural switch costs lead to worse behavior in those with comorbid conditions. Taken together, the relative impact of both worse glycemic control and the presence of comorbidities seems to be widespread, suggesting that better disease management may prevent worse cognitive and neural outcomes. These findings suggest that it is important to separate the effects specific to the disease processes from those that are due to comorbid conditions, particularly since the effects relative to comorbidity status showed specific relationships with behavior.

While this study advances the field’s understanding of the impact of type 2 diabetes and associated comorbid conditions on higher order cognition and the underlying neurophysiology, some limitations need to be acknowledged. These include the characterization of comorbid conditions (yes/no) and the use of HbA_1c_ as a coarse measure of glycemic control. Future studies should examine these variables more in depth to further clarify their causal links to the neural deficits observed. In closing, we found distinct effects of comorbid conditions and glycemic control level on neural switch costs during an executive function task in people with type 2 diabetes. The dynamics of these response differences were largely found within the gamma band, with aberrations in higher frequency dynamics linked to both comorbid conditions and worse glycemic control, although in different brain regions. Neural switch cost differences were related to behavioral switch costs, with the directionality of effects separable by comorbidity status.

These findings add to the growing body of literature illustrating the cognitive decrements and neural dysfunction associated with type 2 diabetes, but are novel in that they identify the separable neurophysiological signatures of comorbidity status and glycemic control level. Future studies should expand on the nature of these effects, including examining whole brain connectivity dynamics, cross-frequency interactions, and structure-function relationships, which may further elucidate the complex nature of how type 2 diabetes affects the brain. Examining individual differences in disease management, comorbid conditions, age, BMI, and other relevant factors and their independent and synergistic effects on the brain may also inform future studies, as the heterogeneity of pathological features and expression may impact cognitive and neural outcomes.

## METHODS

### Participants

Fifty-four participants with type 2 diabetes (25 without and 29 with significant comorbid conditions) were recruited from the Diabetes Clinic at the University of Nebraska Medical Center (UNMC; age range: 55–73 years, 33 females). Participants had to be formally diagnosed with type 2 diabetes for at least 1 year prior to entering the study. Participants with additional complications and comorbidities were considered as such if any of the following conditions were met: (1) nephropathy, as characterized by glomerular filter rate of 45–60 and/or microalbuminuria of 30–300, (2) retinopathy, excluding proliferative retinopathy, (3) peripheral neuropathy, confirmed by documented symptoms or monofilament testing, and/or (4) cardiovascular disease, except recent major cardiovascular events (heart attack, stroke) in the previous 6 months from enrollment. Exclusionary criteria included: (1) any medical diagnosis affecting brain function (e.g., psychiatric and/or neurological disease), (2) known brain neoplasm or lesion, (3) history of cerebrovascular events (i.e., CVA, stroke, TIA) based on previous diagnosis and chart review, (4) history of significant head trauma, seizures or epilepsy, (5) current substance use disorder within the past 6 months, (6) pregnancy or lactation, (7) hospitalization within the previous 90 days, (8) any type of cancer diagnosis or treatment in the past 5 years, except skin cancer, (9) uncontrolled hypertension, with blood pressures greater than 140/90 or 160/100 if currently on medication treatment, (10) body mass index of greater than or equal to 40, (11) liver disease (AST or ALT > 3× normal), (12) any untreated thyroid or B12 deficiencies, (13) history of brain radiation, (14) treatment with antipsychotics, antidepressants, and related medications known to affect brain function, with the exception of as-needed antidepressants following a 24 hour washout period, and (15) ferromagnetic implants.

To study the long-term effects of type 2 diabetes on the brain in a more controlled manner, relative euglycemia (70 to 200 mg/dl) in these participants at the time of study was a prerequisite for participation, measured by point-of-care device prior to study commencement. Written informed consent was obtained from each participant following the guidelines of UNMC’s Institutional Review Board, who approved the study protocol, in accordance with the Declaration of Helsinki.

Prior to MEG, participants underwent a panel of blood tests according to the standards of care described by the American Diabetes Association [48]. Furthermore, demographic and medical history data were collected. For full lab results and general characteristics of the patient groups, see Table 1. Participants then completed cognitive tasks while undergoing MEG, to probe the neurophysiological changes related to essential cognitive processes in this sample.

### Task switch paradigm

During the MEG session, participants were seated in a nonmagnetic chair and instructed to fixate on a crosshair presented centrally for 2600–2800 ms. Following fixation, participants were shown a single centrally-presented integer from 1–4 or 6–9 for 2500 ms, surrounded by either a square or a diamond shape which cued the participant to the appropriate rule set for the trial. If the number was surrounded by a square, the participant had to respond by button press whether the number was less than (index) or greater than (middle) five. If the number was surrounded by a diamond, the participant had to respond by button press whether the number was odd (index) or even (middle). Each rule set occurred 50% of the time, with a pseudo-randomized trial order to have about 50% of all trials repeat the previous rule set (No-Switch) and the remaining trials change rule set from the previous trial (Switch). This ensured participants could not anticipate whether any particular trial would switch or not from the previous rule set. Each trial was 5200 +/− 100 ms, with a total of 200 trials (see Figure 1A). This task has been previously validated by our group [19]. Behavioral metrics were collected concurrently with MEG data, including reaction time and accuracy. Behavioral switch costs, or the difference (i.e., increase) in reaction time between Switch and No-Switch conditions, were computed to characterize the cognitive control component of the task more directly.

### MEG methods and analyses

MEG acquisition and analysis methodology followed standardized pipelines, corresponding to MEG studies previously published by our group [19]. Briefly, MEG data were recorded using a 306-sensor Elekta Neuromag MEG system (Elekta/MEGIN, Helsinki, Finland), sampled at 1 kHz with an acquisition bandwidth of 0.1 – 330 Hz. Each participant’s data were corrected for head motion and subjected to noise reduction using the signal space separation method with a temporal extension [49]. Each participant’s MEG data were then coregistered with structural T1-weighted MRI data. Blink and cardiac artifacts were removed by signal-space projection [50].

The continuous time series was divided into 5000 ms epochs, with a 400 ms baseline directly preceding stimulus onset (−400 to 0 ms). Epochs were rejected using a fixed threshold method, supplemented with visual inspection. The distribution of amplitude and gradient was examined per participant across all trials, and the epochs with highest extrema amplitude and/or gradient values relative to that individual’s distribution were rejected with fixed amplitude and gradient thresholds. Cutoffs were determined individually so as to minimize bias due to head size, sensor proximity, and related variables that influence the signal amplitude, since magnetic field strength decreases exponentially with distance from the source. When appropriate, artifact-free trials in some participants were randomly omitted to balance the total number of trials across groups and/or conditions, which ensures results are not confounded by signal-to-noise ratio differences arising from imbalances in the number of trials. Only correctly answered trials were included in final analyses and participants with poor performance (i.e., near chance accuracy) were excluded from final analyses. The resulting artifact-free trials were transformed in the time-frequency domain using complex demodulation, and the resulting spectral power estimations per sensor were averaged over trials to generate time-frequency plots of mean spectral density. Sensor-level data were normalized by dividing the power of each time-frequency bin by the mean baseline power. Statistical analysis of the sensor-level spectrograms comparing the active window relative to the baseline period across the array of gradiometers during the task period were then computed to determine time-frequency windows to be examined in source-space.

Each data point in the spectrogram was initially evaluated using a mass univariate approach based on the general linear model (GLM), and then corrected for multiple comparisons in stage two. First, *t*-tests were conducted on each data point against the mean baseline value at that frequency and the output spectrograms of *t*-values were thresholded at *p* < 0.05 to define time-frequency bins containing potentially significant oscillatory deviations across all participants. In stage two, time-frequency bins that survived the threshold were clustered with other significant temporally and/or spectrally neighboring bins, and a cluster value was derived by summing the *t*-values of all data points in the cluster. Nonparametric permutation testing was then used to derive a distribution of cluster-values and the significance level of the observed clusters were tested directly using this distribution [51, 52]. From these analyses, time-frequency windows with significant oscillatory responses across all participants and conditions were then imaged at a 4.0 × 4.0 × 4.0 mm resolution using a linearly-constrained minimum variance vector beamformer [53, 54], as implemented in the Brain Electrical Source Analysis (BESA) software (v 7.1). This beamformer method uses spatial filters in the time-frequency domain to calculate source power for the entire brain volume. Following convention, we computed noise-normalized source power per voxel in each participant using active (i.e., task) and passive (i.e., baseline) periods of equal duration and bandwidth. As mentioned above, the passive period was defined as the 400 ms pre-stimulus baseline period (i.e., −400 to 0 ms). The resultant images are referred to as pseudo-*t* maps, with units that reflect noise-normalized power differences at each voxel. Each participant’s functional MEG images were transformed into standardized space using the transform that was previously applied to the structural images and spatially-resampled.

Oscillatory responses that were longer than the baseline in duration (>400 ms) were broken down into non-overlapping time windows of 400 ms or less and imaged separately using the same baseline. These images were then averaged across all relevant windows comprising the oscillatory response to derive a single map per oscillatory response per participant. These whole brain maps were then averaged across all participants to determine the anatomical basis of the oscillatory responses identified in the sensor-level analyses. Next, the whole brain maps of activity were subtracted (switch – no-switch) per individual to compute neural switch costs, or the increased amount of recruitment required to switch rule sets, per oscillatory response. *T*-tests for differences between patients with and without comorbid conditions were computed for each time-frequency window and all output statistical maps of the neural switch cost were thresholded at *p* < 0.01, using a spatial extent threshold (i.e., cluster restriction; k = 20 4 mm^3^ voxels) based on the theory of Gaussian random fields [55]. Significant peaks from these *t-*tests were extracted for post-hoc analyses that focused on correlations with behavioral switch costs. A follow-up analysis of covariance (ANCOVA) was also computed to test the interaction between neural and behavioral switch costs by group, to determine whether the directionality of effects was driven by the presence or absence of comorbid conditions. Further, to examine the specific effects of glycemic control level on task switching in the brain, a whole brain correlational approach was used, with HbA_1c_ as our measure of glycemic control.
